# Growth of preference for solitude in urban and rural Chinese adolescents

**DOI:** 10.3389/fpsyt.2023.1151534

**Published:** 2023-07-06

**Authors:** Xi Chen, Xinxin Sun, Xuan Wu, Junsheng Liu, Dan Li, Xinyin Chen

**Affiliations:** ^1^School of Psychology and Cognitive Science, East China Normal University, Shanghai, China; ^2^Shanghai Key Laboratory of Mental Health and Psychological Crisis Intervention, Shanghai, China; ^3^Shanghai Changning Mental Health Center, Shanghai, China; ^4^Department of Psychology, Shanghai Normal University, Shanghai, China; ^5^Graduate School of Education, University of Pennsylvania, Philadelphia, PA, United States

**Keywords:** preference for solitude, adolescents, social-cultural context, urban, rural, China

## Abstract

**Introduction:**

As individuals enter adolescence, their preference for solitude (PFS) increases with age, which may be a result of balancing the need for social affiliation and the need for autonomy and independence. These needs are shaped by the social-cultural contexts, and thus the growth rate of PFS may differ across social-cultural contexts.This study examined to what extent the developmental trajectory of PFS differed between urban and rural Chinese adolescents.

**Methods:**

Adolescents in urban (n = 326,168 boys, Mage =12.00 years, SD = 0.61) and rural (n = 449, 198 boys, Mage =11.82 years, SD = 0.58) regions in China reported their PFS and shyness each year from Grade 6 to Grade 8. Longitudinal measurement invariance of PFS was established between the urban and rural samples. Location and gender differences in the intercept and the slope of PFS were examined using a latent growth model, while controlling for shyness at each time point.

**Results:**

The analyses revealed that adolescents in both urban and rural regions showed an increasing trajectory of PFS. Although urban and rural adolescents did not differ in the initial level of PFS at Grade 6, urban adolescents’ PFS increased faster than that of the rural adolescents. The urban-rural difference in the slope of PFS remained significant after controlling for the associations between the intercept and the slope of PFS and shyness at each time point. In addition, in the rural region, boys showed a faster increase of PFS than girls, yet gender differences in the initial level of PFS and the developmental trajectory in the urban region were nonsignificant.

**Discussion:**

The findings reveal a normative increasing trend of PFS during early adolescence and faster increase for urban than rural adolescents. To promote adolescents’ psychological well-being, parents, teachers and practitioners need to help adolescents establish a good balance between social interaction and solitude. When deciding what is a good balance, it is important to consider the social-cultural context.

## Introduction

Preference for solitude (PFS) refers to individuals’ tendency to engage in and enjoy solitary activities over being with others (1, 2). Individuals with high PFS have relatively low social approach motivation but do not necessarily feel lonely when alone or anxious in social interaction. As individuals enter adolescence, their PFS increases with age (3–5). There have been arguments regarding the implications of PFS for adolescents. While higher levels and faster increase of PFS was found to be associated with negative peer experience and adjustment outcomes, such as peer victimization, depression, emotion dysregulation, and lower self-esteem (5–7), a few recent studies revealed positive implications of PFS, especially when it is driven by intrinsic enjoyment of solitary activities (4, 8). Thus, understanding factors that contribute to the normative growth of PFS during adolescence helps us interpret its meaning and decide whether or under what conditions we need to be concerned with such growth.

Adolescents may seek more solitude due to various reasons (5, 8), and one of these reasons may be their increasing need for autonomy and independence (9–12). The normative growth of PFS during adolescence may be a result of balancing the need for social affiliation and the need for autonomy and independence. If so, given that these needs may be shaped by the social-cultural contexts (13–15), one would expect differences in the growth rate of PFS across different social-cultural contexts. Examining this social-cultural difference can help us better understand the phenomena of increasing PFS during adolescence.

China has traditionally been a group-oriented society, where individual autonomy is less emphasized than interdependent social relationships. PFS was found to be associated with more adjustment difficulties for Chinese than Western adolescents (16). In the past several decades, with the development of the market economy and the introduction of Western values, individual autonomy is increasingly endorsed by Chinese parents and children (17), and this change happens faster in urban than in rural regions (18, 19). Thus, urban adolescents’ need for autonomy and independence may increase faster than that of rural adolescents. In the current study, we examined whether urban adolescents showed more rapid growth of PFS than rural adolescents in China.

### Developmental trajectory of PFS in adolescence

Adolescence is a unique developmental period to study PFS. On the one hand, individuals spend more and more time with peers from middle childhood to late adolescence (20). On the other hand, as individuals enter adolescence, they may have increasing need for personal space and may voluntarily use their time in solitude for creative activities, emotional regulation and identity development (10–12). Establishing a balance between the need for social affiliation and the increasing need for independence and autonomy is an important developmental task for adolescents (21, 22). The normative growth of PFS may reflect how such a balance change during adolescence.

Age differences in PFS have been examined primarily in Western countries in cross-sectional studies. These studies have shown that adolescents spent more time in solitude than preadolescents (23) and older adolescents had a more positive attitude toward solitude than younger adolescents (11, 24, 25). Fewer studies have examined the developmental trajectory of PFS longitudinally. For example, a study following a U.S. sample from kindergarten to Grade 12 found an increasing trajectory of PFS, especially after children enter Grade 6 (5). Another study following a Flemish sample from 15 to 18 years of age also found an increasing trajectory of positive attitude toward solitude (3). Less is known about the development of PFS in non-Western countries, such as China. A recent study found that Chinese adolescents reported increasing enjoyment in solitude from Grade 7 to Grade 9 (4). Taken together, these studies suggest a normative increase of PFS during adolescence. To what extent the increasing rate of PFS differ across social-cultural contexts remains to be examined.

### The role of social-cultural contexts in the development of PFS

Adolescents’ increasing need for independence and autonomy may be more salient in self-oriented contexts than in group-oriented contexts (26). In more self-oriented contexts, such as in Western countries and urban regions in China, individuals are more likely to experience themselves as independent and distinct from others, whereas in more group-oriented contexts, such as rural regions in China, individuals are more likely to experience themselves as enmeshed in families, communities and work groups (13–15). Thus, in more self-oriented contexts, as individuals enter adolescence, they may be driven by a stronger desire to gain autonomy and establish unique identity. This is supported by previous studies showing that U.S. adolescents showed a faster increase in decision-making autonomy than did Chinese adolescents from Grade 7 to Grade 8 (26) and that urban Chinese third-to-sixth graders were reported by their peers as more assertive than their rural counterparts (18). Align with these findings, adolescents in more self-oriented social-cultural contexts may also show faster increase of PFS.

In addition, meanings of PFS may differ between self-oriented and group-oriented contexts. In more self-oriented contexts, PFS may be viewed as a personal choice and an indicator of self-sufficiency (2). In more group-oriented contexts, individuals are expected to inhibit the expression of their own needs, attend to others’ needs and contribute to the collective welfare (13–15), so PFS may be viewed as more problematic and elicit negative reactions from peers, teachers and parents (16, 27). Given that social evaluations and responses serve as important feedback to shape individuals’ developmental patterns (28), negative social reactions to PFS in more group-oriented contexts may restrain the normative growth of PFS during adolescence.

Only a handful of empirical studies have examined the associations between social-cultural contexts and the development of PFS. A cross-cultural study found that higher PFS was associated with lower peer preference, academic achievement, self-worth and higher loneliness more strongly among Chinese fourth to eighth graders than their Canadian counterparts (16). A more recent study examined PFS among non-migrant and migrant fourth to seventh graders in a Chinese urban region (27). Migrant children moved from rural regions to the urban region and were supposed to hold more group-oriented values than non-migrant urban children. Although the mean levels of PFS did not significantly differ between non-migrant and migrant adolescents, higher PFS was associated with lower peer preference and leadership status more strongly among migrant than non-migrant adolescents. These two studies show that PFS is associated with adjustment difficulties more strongly in more group-oriented contexts. The more negative meanings of PFS in group-oriented contexts may restrain the normative increase of PFS during adolescence in those contexts.

Another line of research has revealed that people in more group-oriented cultures reported higher levels of loneliness than those in the more self-oriented cultures (29, 30). According to the culture-loneliness framework (31), although people in more-group oriented cultures are less likely to be physically isolated compared with those in more self-oriented cultures, they may be more likely to perceive isolation. Because interdependent relationships and conformity to groups are more emphasized in group-oriented cultures, people in these cultures may internalize higher standards regarding ideal social connections, perceive greater discrepancies between ideal and actual social connections, and thus experience higher loneliness. In addition, according to the evolutionary theory of loneliness (32, 33), loneliness may serve as a warning signal and motivates people to repair their insufficient social connections. Thus, experience of higher levels of loneliness may motivate people in group-oriented cultures to seek for more social connections rather than isolation. Adolescents in more group-oriented contexts may also experience more loneliness than those in more self-oriented contexts, and thus may show slower increase in their PFS. Yet, to our knowledge, there has not been any study examining to what extent the developmental trajectory of PFS differs depending on social-cultural contexts.

### The current study

The core aim of this study is to investigate to what extent the developmental trajectory of PFS during adolescence differ across social-cultural contexts. With social changes in China, families and children adopt increasingly self-oriented values, and these changes happen faster in urban regions than in rural regions (18, 19). Thus, we compared the developmental trajectory of PFS among Chinese adolescents in urban and rural regions. Sixth graders were followed for 3 years and reported their PFS each year to obtain their developmental trajectory of PFS. We decided to examine the developmental trajectory starting from Grade 6 because this is the period when PFS began to show faster growth in previous studies (5, 11, 23). We hypothesized that urban adolescents would show higher initial levels and faster growth of PFS than rural adolescents. In addition, given mixed findings regarding gender difference in the development of PFS [see higher PFS in boys than girls in (5, 16), higher enjoyment of solitude in girls than boys in (4), and nonsignificant gender difference in (27)], we also explored gender difference and the interaction between gender and location (urban vs. rural) without specific hypotheses.

In addition, we conducted follow-up analyses to examine whether the urban-rural differences in the development of PFS remained significant after controlling for shyness at each time point, because shyness is closely related to PFS and its prevalence and meaning also differ across social-cultural contexts. Specifically, shyness is a dimension of social withdrawal driven by different motivations compared with PFS (34). Shy children have relatively high social approach motivation, but feel anxious when interacting with others, especially with unfamiliar people (35). Shyness may be more accepted in group-oriented contexts than in self-oriented contexts. Compared with their urban counterparts, rural or migrant children and adolescents in China show higher levels of shyness, and for them shyness is associated with negative developmental outcomes to a less extent (27, 36). After controlling for shyness, PFS more purely reflects low social approach motivation, and we would be able to examine social-cultural differences in adolescents’ development of PFS more rigorously.

## Method

### Participants

Data used in this study originate from two comprehensive longitudinal studies of the psychological and social adjustment of adolescents in mainland China [(blinded for review)]. Participants in the urban group were 326 adolescents (168 boys, *M*_age_ = 12.00 years, SD = 0.61) from Shanghai, an international megacity in East China with top economic strength. Participants in the rural group were 449 adolescents (198 boys, *M*_age_ = 11.82 years, SD = 0.58) with rural registration status in Xuancheng, Anhui province. Xuancheng is a prefecture-level city in East China with moderate economic strength and about 40% of the population reside in rural regions. The participants were recruited from regular public schools randomly selected in the two regions. The regular public schools serve students within the surrounding residential areas rather than select students based on their academic performance or special talent.

At the first time point (Time 1), the adolescents were at Grade 6. Among the adolescents, 59.8 and 45.0% from the urban and rural groups, respectively, were only children, while the rest had one or more siblings. The majority of the adolescents, 78.8 and 73.3% in the urban and rural groups, respectively, were living with both parents, 5.8 and 3.1% living with one parent and others (e.g., step parent), 5.5 and 12.0% living with one parent, 1.8 and 10.5% living with someone other than a parent, and 8.0 and 1.1% did not report this information. Among the parents, 67.8% of the fathers and 63.8% of the mothers in the urban group, 63.9% of the fathers and 48.8% of the mothers in the rural group, had completed junior middle school or higher levels of education.

### Procedure

Data were collected in May and June of each year from 2013 to 2015 in Shanghai, and from 2012 to 2014 in Anhui, respectively. At each time point (Time 1 = Grade 6, Time 2 = Grade 7, Time 3 = Grade 8), participants completed questionnaires regarding their PFS, shyness and demographic information. Participants completed the questionnaires in a group setting at school led by a team of undergraduate and graduate students majored in psychology. Prior to data collection, approvals from the schools and written informed consent from the parents were obtained.

### Measures

#### Preference for solitude

PFS was assessed using the Chinese version of the Child Social Preference Questionnaire [CSPQ, (34)]. We focused on the unsociable subscale, which included seven statements pertaining to preference for spending time alone (e.g., “if given a choice, I prefer to play alone than with other kids,” “I usually prefer doing things alone”) rated on a 5-point scale (1 = never; 5 = always). Good psychometric properties and construct validity of CSPQ has been demonstrated in samples of Chinese children and adolescents (34). Internal reliability of this questionnaire in the current study was acceptable, as indicated by *Cronbach’s* α ranging from 0.876 to 0.925 in the urban group and from 0.856 to 0.902 in the rural group across the time points.

#### Shyness

Shyness was assessed using a modified Chinese version of the Children’s shyness Questionnaire (CSQ, (37), (38)), which includes 12 statements pertaining shyness (e.g., “I feel shy when I have to read aloud in front of the whole class”) rated on a 3-point scale (1 = No; 3 = Yes). The measure was found to be reliable and had good construct validity in the Chinese context (34). Internal reliability of this questionnaire in the current study was acceptable, as indicated by *Cronbach’s* α ranging from 0.834 to 0.865 in the urban group and from 0.771 to 0.844 in the rural group across the time points.

#### Statistical analysis plan

Analyses were carried out in three steps. First, descriptive statistics, correlations of PFS across all time points in urban and rural groups and patterns of missing data were examined. Second, measurement invariance of PFS across time points and between the two groups was evaluated by fitting and comparing a series of sequentially more constrained models. Finally, three latent growth models were fitted to address the main research questions. Two separate models for the urban and rural groups were first fitted to examine the developmental trajectory of PFS and gender as a predictor of the intercept and slope of PFS. Then a main model was fitted to examine location (i.e., urban vs. rural), gender and the interaction between location and gender as predictors of the intercept and slope of PFS. In the follow-up analyses, we first examined measurement invariance of shyness across time points and between the two groups. Then, we fitted the main model when controlling for the association between PFS and shyness and the effect of location and gender on shyness. The Chi-square test, comparative fit index (CFI), Tucker-Lewis index (TLI), and root-mean-square error of approximation (RMSEA) were employed to assess model fit. To be considered acceptable, model fit had to meet the criteria of CFI ≥ 0.90, TLI ≥ 0.90, and RMSEA ≤0.08 (39).

## Results

### Preliminary analyses

Descriptive statistics of PFS for boys and girls in the urban and rural groups at each time point are presented in Table 1. Independent *t*-tests showed that PFS did not significantly differ between the urban and rural groups or between boys and girls in each group at each time point. Bivariate correlations between PFS in the urban and rural groups at each time point are presented in Table 2.

**Table 1:** Means and standard deviations of preference for solitude.

	Urban			Rural		
	Average	Boys	Girls	Average	Boys	Girls
PFS Time 1	2.21 (1.00)	2.19 (1.11)	2.23 (0.89)	2.29 (0.90)	2.20 (0.95)	2.35 (0.86)
PFS Time 2	2.36 (1.03)	2.33 (1.06)	2.40 (1.00)	2.30 (0.91)	2.30 (0.95)	2.30 (0.88)
PFS Time 3	2.49 (1.01)	2.50 (1.10)	2.49 (0.93)	2.38 (0.89)	2.42 (0.96)	2.36 (0.85)

PFS, Preference of Solitude.

**Table 2:** Bivariate correlations between preference for solitude at different time points.

	1	2	3
1. PFS Time1	–	0.476***	0.392***
2. PFS Time2	0.531***	–	0.586***
3. PFS Time3	0.485***	0.612***	–

PFS, Preference of Solitude. Correlations for the urban and rural groups are above and below the diagonal, respectively. ***p < 0.001.

The percentage of missing data were 8.7 and 35.8% at Time 2, 28.4 and 39.4% at Time 3 for the urban and rural groups, respectively. The Little’s missing completely at random (MCAR) test (40) indicated that data were MCAR, χ^2^(9) = 9.032, *p* = 0.434. Independent sample *t*-tests showed no significant difference in PFS at Time 1 between children who participated at both Time 1 and Time 2 (*M* = 2.22, SD = 0.94) and those who were missing at Time 2 (*M* = 2.34, SD = 0.96), *t*(744) = 1.622, *p* = 0.105. Similarly, no significant difference was found in PFS at Time 2 between children who participated at both Time 2 and Time 3 (*M* = 2.32, SD = 0.95) and those who were missing at Time 3 (*M* = 2.39, SD = 1.04), *t*(558) = 0.575, *p* = 0.565. We also conducted these analyses separately for the urban and rural groups, and the results were consistent between the two groups. We handled missing data using the full information maximum likelihood (FIML) estimation.

### Measurement invariance for PFS

As presented in Table 3, scalar measurement invariance of PFS across time points between the urban and rural groups was established, after allowing residuals of two items to covary (“I enjoy being by myself” and “I like spending time alone in my room”).

**Table 3:** Tests of longitudinal measurement invariance between the urban and rural groups for preference of solitude.

Model	*χ* ^2^	df	RMSEA	CFI	TLI	*χ* ^2^	Δ*χ*^2^	Δdf	*p*
**Model fit before allowing residuals of items to covary**									
M1	887.865	372	0.060	0.904	0.891	–	–	–	–
M2	915.866	390	0.059	0.902	0.894	M2 vs. M1	26.377	18	0.091
M3	950.390	411	0.058	0.899	0.897	M3 vs. M2	31.890	21	0.061
**Model fit after allowing residuals of two items to covary** ^a^									
M1	741.279	366	0.051	0.930	0.919				
M2	767.888	384	0.051	0.928	0.921	M2 vs. M1	25.138	18	0.121
M3	803.364	405	0.050	0.926	0.923	M3 vs. M2	34.331	21	0.033

M1, Configural invariance; M2, Metric invariance; M3, Scalar invariance.

a Residuals of the two items “I enjoy being by myself” and “I like spending time alone in my room” were allowed to covary according to model modification index.

### Main models

Results of latent growth models are presented in Table 4. The model for the urban group revealed an increasing slope of PFS from Grade 6 to Grade 8, and the intercept and slope of PFS did not differ between boys and girls. The model for the rural group also revealed an increasing slope of PFS, and boys showed faster growth of PFS than girls, although boys and girls did not differ in their initial levels of PFS at Grade 6. The main model revealed that adolescents in the urban group showed faster growth of PFS than adolescents in the rural group, whereas the intercept of PFS did not significantly differ between the two groups. Gender and the interaction between gender and location did not significantly predict the intercept or slope of PFS.

**Table 4:** Latent growth models for preference for solitude.

	Unstandardized results			Standardized results		
	Estimate	SE	*p*	Estimate	SE	*p*
**Model for the urban group (*n* = 326)**xs						
Intercept (I)	2.18 (0.475)	0.09	<0.001	2.86	0.29	<0.001
Slope (S)	0.16 (0.071)	0.05	0.004	0.42	0.15	0.006
I-S covariance	−0.08	0.07	0.242	−0.28	0.17	0.099
Gender→ I	0.06	0.11	0.587	0.04	0.07	0.589
Gender→ S	−0.00	0.07	0.974	−0.00	0.10	0.974
Model fit: *χ*^2^ (2) = 0.218, *p* = 0.897, CFI = 1.000, TLI = 1.022, RMSEA = 0.000						
**Model for the rural group (*n* = 449)**						
Intercept (I)	2.21 (0.581)	0.07	<0.001	3.18	0.29	<0.001
Slope (S)	0.12 (0.140)	0.05	0.007	0.44	0.21	0.031
I-S covariance	−0.04	0.05	0.362	−0.23	0.18	0.206
Gender→ I	0.14	0.09	0.109	0.10	0.06	0.108
Gender→ S	−0.11	0.05	0.044	−0.20	0.11	0.070
Model fit: *χ*^2^ (2) = 0.486, *p* = 0.784; CFI = 1.000, TLI = 1.022, RMSEA = 0.000						
**Model examining location as a predictor of the growth curve (*N* = 775)**						
Intercept (I)	2.19 (0.518)	0.05	<0.001	3.04	0.21	<0.001
Slope (S)	0.14 (0.102)	0.04	<0.001	0.43	0.12	<0.001
I-S covariance	−0.06	0.04	0.148	−0.25	0.12	0.046
Location → I	−0.05	0.04	0.270	−0.07	0.06	0.268
Location → S	0.07	0.03	0.010	0.22	0.09	0.013
Gender→ I	0.10	0.07	0.156	0.07	0.05	0.158
Gender→ S	−0.06	0.05	0.207	−0.09	0.07	0.208
Location × Gender→ I	−0.04	0.07	0.598	−0.04	0.07	0.598
Location × Gender→ S	0.05	0.05	0.242	0.11	0.10	0.256
Model fit: *χ*^2^ (4) = 0.683, *p* = 0.953; CFI = 1.000, TLI = 1.000, RMSEA = 0.000						

Location coded as Urban = 1, Rural = −1. Gender coded as Boy = 0, Girl = 1. Residual variance of the intercepts and slopes are reported in parenthesis. Residual variance of the intercept and slope did not differ between the urban and the rural groups.

### Follow-up analyses

**Figure 1:**
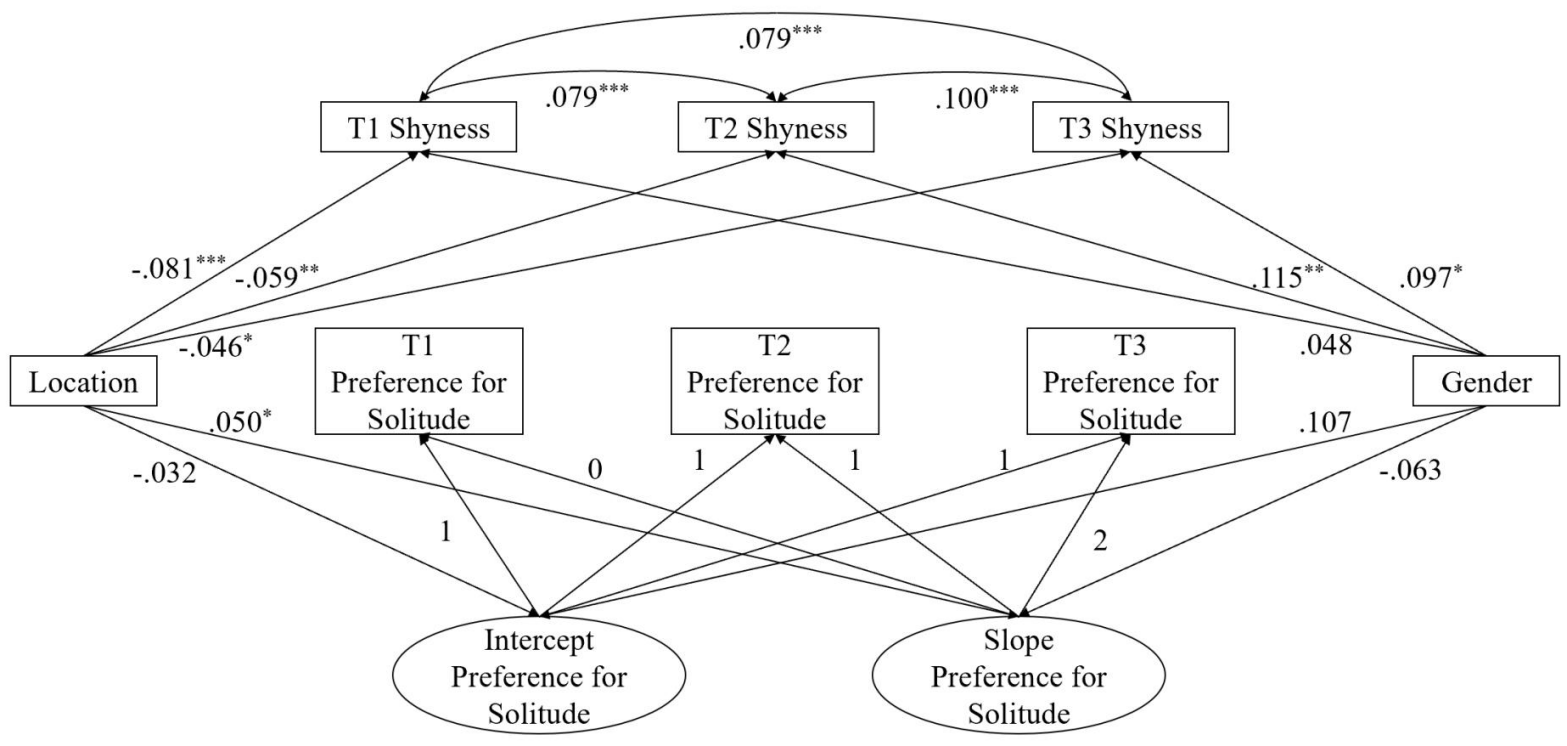
Latent growth model of preference for solitude (PFS). *p < 0.05, **p < 0.01, ***p < 0.001. Location coded as Urban = 1, Rural = −1. Gender coded as Boy = 0, Girl = 1. T1 = Time 1, T2 = Time 2, T3 = Time 3. The covariances between the intercept and the clope of PFS and shyness at each time point were estimated but not shown in the figure for ease of presentation. Model fit: *χ*^2^ (6) = 1.912 *p* = 0.928; CFI = 1.000, TLI = 1.000, RMSEA = 0.000.

Longitudinal measurement invariance of shyness cannot be established given the poorly fitted models assuming configural measurement invariance at the three time points for both the urban and rural groups. Thus, we were not able to estimate the latent growth curve for shyness. Yet, configural, metric and scalar invariance for shyness at each time point between the urban and rural groups were established (see Table 5). Thus, we estimated the covariance between the intercept and the slope of PFS and shyness at each time point, as well as the predictive effects of location and gender on shyness at each time point (see Figure 1). In this follow-up analysis, urban adolescents continued to show faster increase of PFS than rural adolescents, and their PFS at Grade 6 did not differ significantly. In addition, rural adolescents reported higher shyness than urban adolescents at each time point, and girls reported higher shyness than boys at Grades 7 and 8. Shyness at each time point was positively related to the intercept of PFS, and higher shyness at Grade 6 was related to slower increase of PFS. Shyness at each time point was positively related with each other.

**Table 5:** Tests of measurement invariance at each time point between the urban and rural groups for shyness.

Model	*χ* ^2^	df	RMSEA	CFI	TLI	*χ* ^2^	Δ*χ*^2^	Δdf	*p*
**Model fit at Time 1**									
M1	205.589	108	0.049	0.934	0.920	–	–	–	–
M2	214.773	119	0.046	0.935	0.928	M2 vs. M1	7.792	11	0.732
M3	270.741	131	0.053	0.906	0.905	M3 vs. M2	58.599	12	0.000
**Model fit at Time 2**									
M1	227.802	108	0.063	0.924	0.907	–	–	–	–
M2	237.331	119	0.060	0.924	0.916	M2 vs. M1	7.793	11	0.732
M3	307.145	131	0.069	0.888	0.887	M3 vs. M2	75.171	12	0.000
**Model fit at Time 3**									
M1	212.884	108	0.063	0.931	0.916	–	–	–	–
M2	229.838	119	0.062	0.927	0.919	M2 vs. M1	16.109	11	0.137
M3	282.450	131	0.069	0.901	0.900	M3 vs. M2	55.555	12	0.000

M1, Configural invariance; M2, Metric invariance; M3, Scalar invariance.

## Discussion

In this study, we compared the developmental trajectory of PFS from Grade 6 to Grade 8 among Chinese adolescents in an urban group and a rural group. Consistent with our hypothesis, both urban and rural adolescents showed an increasing trajectory of PFS from Grade 6 to Grade 8, and the growth rate of PFS among urban adolescents was faster than that of the rural adolescents. We suspect that this difference may be driven by different growth rate of adolescents’ need for autonomy and independence in the urban vs. rural regions. As individuals enter adolescence, they may experience a normative increase in their need to gain autonomy and establish a unique identity (21, 22, 26). Driven by this need, adolescents in both urban and rural areas may increasingly seek personal space and appreciate time in solitude. With more rapid social changes happening in urban China, urban adolescents may adopt more self-oriented social values (18, 19), and thus show a faster increase of PFS. In addition, in more group-oriented social contexts, adolescents’ PFS may elicit more negative reactions from peers (16, 27), which may further undermine the normative increase of adolescents’ PFS in rural regions. Furthermore, in light of the culture-loneliness framework (31) and the evolutionary theory of loneliness (32, 33), we also speculate that rural adolescents may experience higher levels of loneliness, which may serve as an alarming signal for them to maintain social connections and contribute to slower increase of PFS. This finding echoes with existing studies showing social-cultural differences in the implications of PFS (16, 27) and provides additional evidence regarding the role of social-cultural contexts in the development of adolescents’ PFS.

Inconsistent with our hypothesis, the initial level of PFS at Grade 6 did not differ between the urban and rural groups. Since PFS begins to show a faster increase as individuals enter adolescence (5, 11, 23), social-cultural difference in the average levels of PFS may take time to emerge. In fact, the comparison of PFS between the two groups at each time point did not reveal significant difference. Previous studies comparing mean levels of PFS between Chinese and Canadian children and between migrant and non-migrant urban Chinese children (age ranging from Grade 4 to Grade 8) did not find significant difference either. It is possible that the social-cultural effect on the development of PFS first demonstrates in different growth rate. With accumulation, difference in the mean levels of PFS may emerge in later adolescence, which merits investigation in future studies.

As to gender difference, we found that boys showed faster growth than girls in the rural group. This finding aligns more with the existing studies showing higher PFS in boys than in girls. For example, Liu et al. (16) found that boys in fourth to eighth grade were reported by their peers as having higher PFS than girls. Ladd et al. (5) found that although boys and girls showed a similar growth trend of PFS from kindergarten to Grade 12, with the accumulation of gender difference, by late adolescence, boys scored higher than girls on PFS. We suspect that the gender difference may be attributed to gender-stereotypical ideologies that boys should be more independent and autonomous, whereas girls are more expected to develop and maintain close social relationships (41, 42). In contrast with the rural group, the gender difference in the urban group was not significant. This may be due to a relatively more egalitarian gender role in urban areas than in rural areas (43, 44). This social-cultural effect on gender difference needs further examination, given the nonsignificant interaction between location and gender in the trajectory of PFS.

After controlling for the associations between shyness and the development of PFS, the urban-rural difference in the growth rate of PFS remained significant. In addition, consistent with existing findings (27, 38), we found that rural adolescents reported higher shyness than urban adolescents at each time point, and girls reported higher shyness than boys at Grades 7 and 8. Although both PFS and shyness contribute to social withdrawal behaviors, they showed distinct associations with the social-cultural context and gender. This may be due to the different motivations for social connections underlying PFS and shyness. Adolescents with high PFS have low social approach motivation, which is more accepted in self-oriented contexts (34). In contrast, shyness reflects high social approach motivation combined with anxious for social evaluation, which is more accepted in group-oriented contexts (27, 38) and aligns with the gender-stereotypical ideology that girls should value close social relationships (41, 42). These findings show the difference between PFS and shyness and provide additional support for our hypothesis regarding urban-rural difference in the development of PFS.

We note several limitations and future directions. First, we only examined the normative developmental trajectory of PFS, yet there are rich individual differences in this trajectory. A study following children’s social withdrawal (a broader concept including both PFS and shyness) from Grade 5 to Grade 8 found three trajectory categories, i.e., a low-stable trajectory, a decreasing trajectory and an increasing trajectory (45). Future studies may examine whether there are sub-groups of adolescents showing qualitatively different developmental trajectories of PFS, and to what extent composition of these sub-groups differ between urban and rural adolescents.

Second, we interpreted the different growth rates of PFS between urban and rural adolescents as due to different cultural values (i.e., self-oriented vs. groups-oriented values) in urban and rural regions, but did not directly analyze adolescents’ cultural values as predictors of their developmental trajectory of PFS. An important future direction is to test to what extent difference in the developmental trajectory of PFS across social-cultural contexts can be explained by difference in the mean-level and developmental trajectory of adolescents’ cultural values. In addition, future studies may examine whether loneliness acts as a mediator between the social-cultural context and the growth of adolescents’ PFS.

Third, related to the previous point, we focused our interpretation on cultural values given existing theoretical (2, 13–15) and empirical (16, 27) work, yet other contextual factors may also contribute to the urban-rural difference in the growth rate of PFS. For example, given that the one-child policy was stricter in urban regions than in rural regions (44), a greater proportion of urban adolescents are only children. Without the company of siblings, only children may have more opportunities to spend time in solitude. To what extent only-children status and other contextual factors, such as having a separate room, may contribute to self-oriented values and the development of PFS needs further investigation.

Fourth, although we inferred the increasing need for independence and autonomy as a factor driving the growth of PFS during adolescence, PFS may be driven by other factors. Solitude may be an active choice due to the intrinsic enjoyment of being alone or a passive reaction to peer rejection or victimization (5, 8). It is an important future direction to explicitly measure the different motivations driving PFS and examine how these motivations jointly contribute to the development of PFS during adolescence.

Finally, the participants were from only two regions and the findings may not be generalizable to other urban and rural regions. The urban participants were from Shanghai, one of the most developed and internationalized cities in China. Adolescents in Shanghai may hold more self-oriented values and show more rapid growth of PFS than adolescents from less developed urban regions. The rural participants were from a region with moderate economic strength in East China and they may show more rapid growth of PFS than adolescents in more remote and less developed rural regions. Thus, findings of the current study need to be replicated in other urban and rural regions in China.

Despite these limitations, this study enriches our understanding about the development of PFS during adolescence by using a longitudinal design and comparing the developmental trajectory across social-cultural contexts. The findings reveal a normative increasing trend of PFS during early adolescence and faster increase for urban than rural adolescents, as well as faster increase for boys than girls in rural regions. The urban-rural difference in the growth of PFS remained significant after controlling for shyness. While excessive PFS may result from negative peer experience and contribute to adjustment difficulties (5, 16, 27), the normative increase of PFS during adolescence may be partially driven by adolescents’ growing need for independence and autonomy and have positive implications for their adjustment (4). In fact, both negative feelings due to not meeting ones’ need for social connections [i.e., loneliness; (46)] and negative feelings due to not meeting ones’ need for solitude [i.e., aloneliness; (47, 48)] have negative implications for individuals’ mental health and adjustment. Thus, to promote adolescents’ psychological well-being, parents, teachers and practitioners need to help adolescents establish a good balance between social interaction and solitude. Considering the faster increase of PFS in urban adolescents than in rural adolescents, it is important to consider the social-cultural context when deciding what is a good balance between social interaction and solitude.

## Data availability statement

The raw data supporting the conclusions of this article will be made available by the authors, without undue reservation.

## Ethics statement

The studies involving human participants were reviewed and approved by East China Normal University. Written informed consent to participate in this study was provided by the participants’ legal guardian/next of kin.

## Author contributions

XiC contributed idea of the manuscript, conducted the main analyses, wrote the Introduction and Discussion, and revised the Method and Results. XS conducted literature review and supplementary analyses. XW conducted descriptive analyses, wrote an initial draft of the Method and Results, and made the initial versions of the tables. JL, XinC, and DL conducted the larger studies, from which data in this manuscript were drawn. All authors contributed to the article and approved the submitted version.

## Funding

This study was supported by the Research Project of Shanghai Science and Technology Commission(20dz2260300), the Fundamental Research Funds for the Central Universities (2020ECNU-HLYT052; 2021ECNU-YYJ019) and the Shanghai Sailing Program (21YF1411300).

## Conflict of interest

The authors declare that the research was conducted in the absence of any commercial or financial relationships that could be construed as a potential conflict of interest.

## Publisher’s note

All claims expressed in this article are solely those of the authors and do not necessarily represent those of their affiliated organizations, or those of the publisher, the editors and the reviewers. Any product that may be evaluated in this article, or claim that may be made by its manufacturer, is not guaranteed or endorsed by the publisher.
